# Tracing the epidemic history of HIV-1 CRF01_AE clusters using near-complete genome sequences

**DOI:** 10.1038/s41598-017-03820-8

**Published:** 2017-06-22

**Authors:** Xingguang Li, Haizhou Liu, Lu Liu, Yi Feng, Marcia L. Kalish, Simon Y. W. Ho, Yiming Shao

**Affiliations:** 1 State Key Laboratory for Infectious Disease Prevention and Control, National Center for AIDS/STD Control and Prevention, Chinese Center for Disease Control and Prevention, Beijing, China; 2 Collaborative Innovation Center for Diagnosis and Treatment of Infectious Diseases, Hangzhou, Zhejiang China; 3 Centre for Emerging Infectious Diseases, The State Key Laboratory of Virology, Wuhan Institute of Virology, University of Chinese Academy of Sciences, Wuhan, China; 4 Shantou University Medical College, Shantou, 515041 China; 5 College of Veterinary Medicine, South China Agricultural University, Guangzhou, 510642 China; 6 Vanderbilt Institute for Global Health, Vanderbilt University School of Medicine, Nashville, Tennessee USA; 7 School of Life and Environmental Sciences, University of Sydney, Sydney, New South Wales 2006 Australia

## Abstract

Human immunodeficiency virus (HIV) has a number of circulating recombinant forms that are the product of recombination between different HIV subtypes. The first circulating recombinant form of HIV-1 to be identified was CRF01_AE, which originated in Central Africa and is now most prevalent in Southeast and East Asia. In this study, we investigated the timescale, evolutionary history, and population genetics of the HIV-1 CRF01_AE strains primarily responsible for the epidemic in Asia. A further aim of our study was to define and standardize the nomenclature and provide well-characterized reference sequences for the phylogenetic transmission clusters of CRF01_AE. We analysed a data set of 334 near-complete genome sequences from various risk groups, sampled between 1990 and 2011 from nine countries. Phylogenetic analyses of these sequences were performed using maximum likelihood and Bayesian methods. Our study confirms that the diversity of HIV-1 CRF01_AE originated in Central Africa in the mid-1970s, was introduced into Thailand between 1979 and 1982, and began expanding there shortly afterwards (1982–1984). Subsequently, multiple clusters significantly contributed to China’s HIV epidemic. A Bayesian skyline plot revealed the rapid expansion of CRF01_AE in China around 1999–2000. We identified at least eight different clusters of HIV-1 CRF01_AE formed by rapid expansion into different risk groups and geographic regions in China since the late 1980s.

## Introduction

Human immunodeficiency virus (HIV) has undergone multiple cross-species transmissions from nonhuman primates into humans, producing two major types^1^: HIV-1 and HIV-2. The globally circulating strains of HIV-1 are extremely diverse, as a result of high rates of mutation, recombination, and replication^2–6^. Group M, the most common group of HIV-1, is responsible for the large majority of AIDS cases across the world. It is further classified into nine subtypes (A–D, F–H, J, and K) and four sub-subtypes (A1, A2, F1, and F2), as well as a number of circulating recombinant forms (CRFs) with various unique recombinant forms (URFs)^7, 8^.

The first CRF of HIV-1 to be identified was CRF01_AE, initially named “subtype E”. It represents a putative recombinant between subtypes A and E, but a parental (non-recombinant) subtype E has not been found^9, 10^. Although CRF01_AE contains a subtype E *vif*, *vpr*, *env*, *nef*, and long terminal repeat (LTR), most or all of the remaining genome derives from subtype A. Although the “subtype E” segments in this CRF should be referred to as “U” (unclassified) according to the recommended nomenclature for HIV-1^8^, the historical “subtype E” designation has been retained to refer to the putative non-A regions in this CRF.

CRF01_AE is most prevalent in Thailand and neighboring countries in Southeast and East Asia. It originated in Central Africa and has been found among mid-1980s samples from the Democratic Republic of Congo^11^. However, the earliest known strains of CRF01_AE were first identified in samples from northern Thailand in 1989 among female commercial sex workers^12–14^. CRF01_AE then spread into various risk groups in Thailand and neighboring regions^15–19^. It is also a component of at least 16 CRFs identified in Africa and Asia (http://www.hiv.lanl.gov).

Viral transmission events can be investigated using phylogenetic analyses of HIV sequences isolated from different patients. An analysis of 33 near-complete genomes found that CRF01_AE in Vietnam formed at least three phylogenetic transmission clusters from founder strains being introduced into new locations and risk groups^17^. Another study identified at least three phylogenetic transmission clusters of strains that most likely contributed to the CRF01_AE epidemic in Hong Kong^20^. A recent analysis of 1957 CRF01_AE *gag p*17 sequences, collected between 1990 and 2010 from 15 different countries, identified 27 phylogenetic transmission clusters^21^. A more comprehensive study used a statistical phylogeographic analysis of 2736 CRF01_AE partial *pol* sequences to uncover global patterns of dispersal^22^. Other phylogenetic studies have shown that the CRF01_AE epidemic in China was driven by multiple independent clusters introduced in the 1990s^18, 23, 24^. Despite this research into CRF01_AE, we still have an incomplete understanding of the distinct clusters circulating in the Asian region.

To obtain a more comprehensive picture of the spatiotemporal dynamics of the HIV-1 CRF01_AE epidemic in Asia, we analysed a data set of 334 near-complete genomes of CRF01_AE sampled from 1990 to 2011 from nine countries. We used phylogenetic, molecular clock, and Bayesian skyline analyses to explore the origin of CRF01_AE transmission clusters and to estimate the timeline and demographic history of each of the clusters. We also suggest the use of consistent and standardized nomenclatural criteria for the transmission clusters and provide a set of 10 well-characterized reference sequences.

## Materials and Methods

### Sample selection and sequence data

Based on the results of an HIV molecular epidemiology survey conducted between 2010 and 2011 of various risk groups in Jilin province, China^25^, we obtained four new near-complete genome sequences of CRF01_AE (552–9636 nt relative to HXB2) from plasma virus RNA as previously described^18, 26–28^. These sequences, from one heterosexual and three men who have sex with men (MSM), were named JL100034, JL100038, JL110010, and JL110056, respectively (GenBank accession numbers KP860667–KP860670).

All available near-complete genome sequences of CRF01_AE (one per patient) with known sampling dates and geographic information were retrieved from the Los Alamos National Laboratory (LANL) HIV Sequence Database (http://www.hiv.lanl.gov). HIV BLAST was used to identify closely related CRF01_AE sequences in the HIV-1 database^29^. Sequence quality was analysed using the Quality Control tool on the LANL site, whereas the genotype assignment of all sequences was confirmed using RIP v.3.0^30^. Hypermutation analysis was performed using Hypermut v2.0^31^. A total of 330 sequences of CRF01_AE were combined with the four newly generated sequences to form this data set (Tables 1, 2 and 3 and S1).

**Table 1:** Geographic source, sampling year, and risk factor for HIV-1 CRF01_AE strains analysed in the present study.

Geographic source	Sampling year	n^a^	Risk factor^b^					
			Hetero	IDU	MSM	MTCT	ST	n/a
China	1997–2011	154 (4)	56 (1)	21	56 (3)	3	9	9
Vietnam	1997–1998	33	17	16				
Afghanistan	2007	1	1					
Central African Republic	1990	3	1					2
Hong Kong	2004	1						1
Indonesia	1993	1						1
Japan	1993–2000	2	2					
Thailand	1990–2009	134	14	30		1		89
United States	1998–2005	5	4					1
**Total**		**334 (4)**	**95 (1)**	**67**	**56 (3)**	**4**	**9**	**103**

^a^The numbers of HIV-1 CRF01_AE sequences newly reported in this study are shown in parentheses.

^b^Risk group: Hetero, heterosexual; IDU, injecting drug user; MSM, men who have sex with men; MTCT, mother-to-child transmission; ST, sexual transmission, unspecified type; n/a, not available.

**Table 2:** Classification and risk factor of distinct HIV-1 CRF01_AE clusters analysed in the present study.

CRF01_AE cluster	n^a^	Risk factor^b^					
		Hetero	IDU	MSM	MTCT	ST	n/a
CRF01_1AE	40 (1)	24 (1)	14				2
CRF01_2AE	26	8	18				
CRF01_3AE	3	1	1				1
CRF01_4AE	25	1	1	21		1	1
CRF01_5AE	37 (3)	2		34 (3)			1
CRF01_6AE	4	4					
CRF01_7AE	3	3					
CRF01_8AE	5	3				1	1
CRF01_9AE	3		3				
CRF01_10AE	5		5				
Ungrouped	183	49	25	1	4	7	97
**Total**	**334 (4)**	**95 (1)**	**67**	**56 (3)**	**4**	**9**	**103**

^a^Numbers of HIV-1 CRF01_AE sequences newly reported in our study are shown in parentheses.

^b^Risk group: Hetero, heterosexual; IDU, injecting drug user; MSM, men who have sex with men; MTCT, mother-to-child transmission; ST, sexual transmission, unspecified type; n/a, not available.

**Table 3:** Classification and sampling year of distinct HIV-1 CRF01_AE clusters analysed in the present study.

CRF01_AE cluster	n^a^	Sampling year				
		1990–1994	1995–1999	2000–2004	2005–2009	2010–2011
CRF01_1AE	40 (1)				38	2 (1)
CRF01_2AE	26		19		7	
CRF01_3AE	3				3	
CRF01_4AE	25				9	16
CRF01_5AE	37 (3)				21	16 (3)
CRF01_6AE	4				4	
CRF01_7AE	3				3	
CRF01_8AE	5				5	
CRF01_9AE	3		2	1		
CRF01_10AE	5		3	2		
Ungrouped	183					
**Total**	**334 (4)**		**24**	**3**	**90**	**34 (4)**

^a^Numbers of HIV-1 CRF01_AE sequences newly reported in our study are shown in parentheses.

An initial alignment of all 334 sequences was performed using Gene Cutter from the LANL site and then adjusted manually in BioEdit v7.0.9.0^32^. If gaps were inserted unambiguously and the alignment columns contained gaps in more than 50% of the sequences, they were removed using Gap Strip/Squeeze v2.1.0 on the LANL site.

The combined data set of 334 sequences includes samples from various risk groups: heterosexuals (Hetero); injecting drug users (IDUs); men who have sex with men (MSM); mother-to-child transmission (MTCT); sexual transmission with unspecified type (ST); and unknown risk. The samples are drawn from broad geographical regions: 13 provinces in China; Afghanistan; Central African Republic; Hong Kong; Indonesia; Japan; Thailand; United States; and five provinces in Vietnam. As listed in Tables 1 and S1, 154 were obtained from various risk groups in 13 provinces across China between 1997 and 2011 and 180 were previously reported from 8 other countries between 1990 and 2009. The main risk groups are unknown risk (30.84%), Hetero (28.44%), IDUs (20.06%), and MSM (16.77%). The samples are primarily from China (46.11%), Thailand (40.12%), and Vietnam (9.88%).

The study was approved by the institutional review board of the National Center for AIDS/STD Control and Prevention, China CDC. A written informed consent, as well as a socio-demographic questionnaire, was obtained for each of the four new near-complete genome sequences. All methods were performed in accordance with the relevant guidelines and regulations.

### Phylogenetic analyses

To study the amount of evolutionary information contained in the data set, a likelihood-mapping analysis^33^ was performed using TREE-PUZZLE v5.3^34^ by analysing 10000 randomly chosen quartets for the entire tree. For each sequence quartet, three unrooted tree topologies are possible. For a random sample of quartets, the likelihoods for the three possible topologies are reported as dots in an equilateral triangle. The distribution of points in different sections of this triangle indicates the tree-likeness of the data: the three corners represent fully resolved tree topologies, indicating the presence of tree-like phylogenetic signal; the center represents the sets of points where all three trees are equally supported, indicating a lack of phylogenetic signal; and the three areas on the sides indicate support for conflicting tree topologies. To infer the phylogeny, we used a maximum-likelihood approach with the GTR + G model in RAxML v8.0.9^35^. Support for the inferred relationships was evaluated by a bootstrap analysis with 1000 replicates.

Strategies for identifying and defining transmission clusters differ between studies. Here we identify them on the basis of within-cluster genetic distance (cut-off of 6%) and bootstrap support (cut-off of 99%) for groupings with more than two sequences, as implemented in Cluster Picker v1.2.3^36^. Genetic distances between and within clusters were calculated in MEGA v7.1.18^37^ using the maximum composite likelihood^38^ with 1000 bootstrap replicates. Rate variation among sites was modelled with a gamma distribution. A plot of genetic distances between clusters was generated using the pheatmap package in R. In addition, we used the web-based tool Evolview v2^39^ to visualize and annotate the phylogenetic tree with geographic location, phylogenetic cluster, and risk group.

To investigate the temporal signal in the data set, analyses of the correlation between root-to-tip genetic distance and year of sampling were performed on the maximum-likelihood tree using the program TempEst v1.5^40^. We also estimated the evolutionary rate for the data set using least-squares dating in LSD v0.2^41^. We then used a Bayesian phylogenetic approach for joint estimation of the ages of each of the 10 CRF01_AE clusters and the demographic history of all of the strains. This was done by analysing the 334 sequences using a GTR + G substitution model with an uncorrelated lognormal relaxed-clock model^42^ and a Bayesian skyline coalescent tree prior^43^ in BEAST v1.8.2^44^. The molecular clock was calibrated using the sampling dates of the sequences. Posterior distributions of parameters, including the tree, were estimated using Markov chain Monte Carlo (MCMC) sampling. The MCMC was run for 500 million steps, with the first 10% removed as burn-in. Samples were drawn every 50,000 steps. Convergence and sufficient sampling were evaluated by calculating the effective sample sizes of the parameters using Tracer v1.5 (http://beast.bio.ed.ac.uk/software/tracer). Trees were summarized as maximum clade credibility (MCC) trees using TreeAnnotator (part of the BEAST package) and visualized in FigTree v1.4.3.

We wished to test the hypothesis that a tip with a given discrete trait (geographic location or risk group) is more likely to share that discrete trait with a neighboring tip than would be expected by chance. For each discrete trait in our data set, we calculated the association index (AI), Fitch parsimony score (PS), and monophyletic clade size (MC) statistics using Bayesian Tip-Significance Testing (BaTS) software version 1.0^45^. AI and PS scores indicate migration events between trait values, and MC scores indicate the number of taxa in the largest clade monophyletic for that trait value. Therefore, low AI and PS scores and high MC scores indicate a strong trait association.

To accommodate the uncertainty in the phylogenetic estimate, we used the posterior set of trees from the Bayesian phylogenetic analysis described above. The topological robustness of this sample of trees was determined by comparing it with the null distribution of trees obtained from 10,000 bootstrap replicates of discrete characters. The *P*-value is then calculated as the proportion of trees from the null distribution for which the value of the statistic is equal to, or more extreme than, the median estimate from the posterior sample of trees. We reject the null hypothesis for significance levels of 0.001, 0.001, and 0.05 for AI, PS, and MC statistics, respectively.

## Results

### Likelihood mapping and phylogeny of HIV-1 CRF01_AE strains

The phylogenetic signal from the data set was investigated by likelihood-mapping analysis^33^. Our likelihood-mapping analysis revealed that the quartets from the data set were primarily distributed in the corners (92.2%) rather than the sides (7.5%) or center (0.3%) of the triangle, indicating a strong tree-like phylogenetic signal (Supplementary Figure S1).

**Figure 1:**
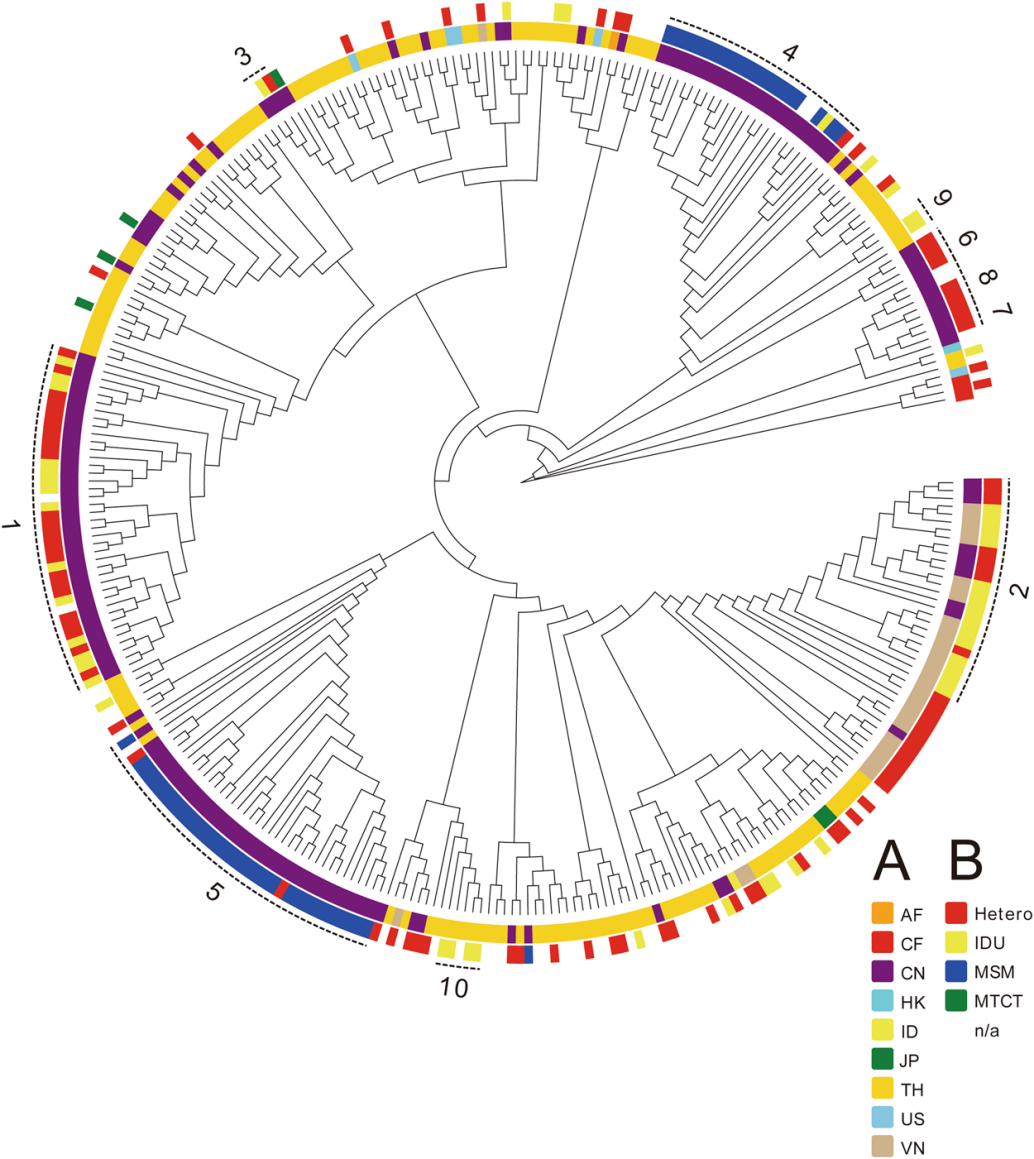
Maximum-likelihood phylogeny of HIV-1 CRF01_AE strains. Maximum-likelihood phylogeny of near-complete genome sequences of HIV-1 CRF01_AE. The two circles of colored cells show geographic location (inner circle, **A**) and risk group (outer circle, **B**).

**Figure. 2:**
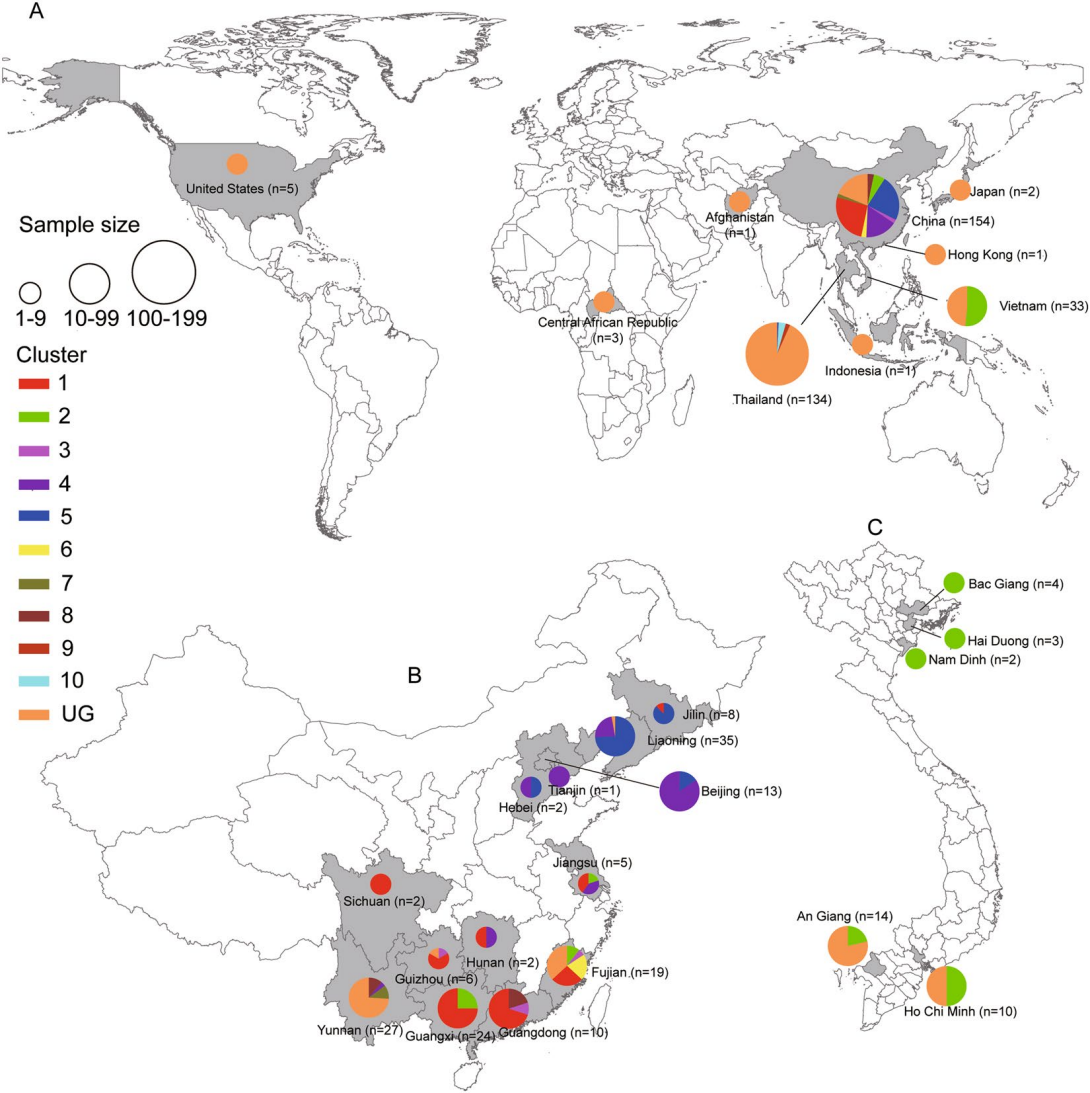
Geographic distribution of HIV-1 CRF01_AE clusters identified in the present study. The geographic distribution of HIV-1 CRF01_AE clusters is shown at the (**A**) country level, and at the provincial level for (**B**) China and (**C**) Vietnam. Each CRF01_AE cluster identified in this study is color-coded, as shown on the left. Maps were obtained from Craft MAP website (http://www.craftmap.box-i.net/).

The phylogeny of HIV-1 CRF01_AE strains, inferred using maximum likelihood, indicates the presence of 10 transmission clusters (Figs 1 and 2 and S2 and Tables 2 and 3 and S1). Cluster names were based on our previous numbering system and with the addition of new clusters in this study^18^. Sequences from Cluster 1 (designated as CRF01_1AE; n = 40) were found among Hetero (n = 24), IDU (n = 14), and unknown risk (n = 2) patients in eight provinces of China. Cluster 2 sequences (CRF01_2AE; n = 17) were found among Hetero (n = 8) and IDU (n = 18) patients in three provinces of China and five provinces of Vietnam. Sequences from Cluster 3 (CRF01_3AE; n = 30) were collected from Hetero (n = 1), IDU (n = 1), and unknown risk (n = 1) patients in three provinces of China. The risk groups associated with these three clusters were primarily Hetero and IDUs.

Cluster 4 sequences (CRF01_4AE; n = 25) were found in seven provinces of China, whereas Cluster 5 sequences (CRF01_5AE; n = 37) were collected from four provinces of China and from Thailand. The predominant risk group associated with Clusters 4 and 5 was MSM. The four sequences from Cluster 6 (CRF01_6AE) were all collected from Hetero patients in Fujian province, China. Cluster 7 (CRF01_7AE) included only three sequences collected from Hetero patients in Yunnan province, China. Cluster 8 sequences (CRF01_8AE; n = 5) were found among Hetero (n = 3), ST (n = 1), and unknown risk (n = 1) patients in two Chinese provinces. Cluster 9 (CRF01_9AE; n = 3) only included sequences collected only from IDUs in Thailand. Cluster 10 (n = 5) included sequences collected only from IDUs in Thailand, with the short branches in the tree implying that these were recent transmissions.

The remaining 183 sequences (designated as Ungrouped) were scattered throughout the main CRF01_AE clade and had been sampled in China (n = 29), Vietnam (n = 16), Afghanistan (n = 1), Hong Kong (n = 1), Indonesia (n = 1), Japan (n = 2), Thailand (n = 125), United States (n = 5), and Central African Republic (n = 3). Of the 56 MSM sequences in our analysis, all but one were found within either CRF01_4AE (n = 21) or CRF01_5AE (n = 34) and all originated from China.

### Genetic diversity and demographic analysis

We estimated the genetic diversity within and between each of the 10 HIV-1 CRF01_AE clusters (Supplementary Figure S4). The smallest genetic distance separated Clusters 2 and 9 (4.4%), whereas the largest was between Clusters 4 and 8 (7.6%).

**Figure 3:**
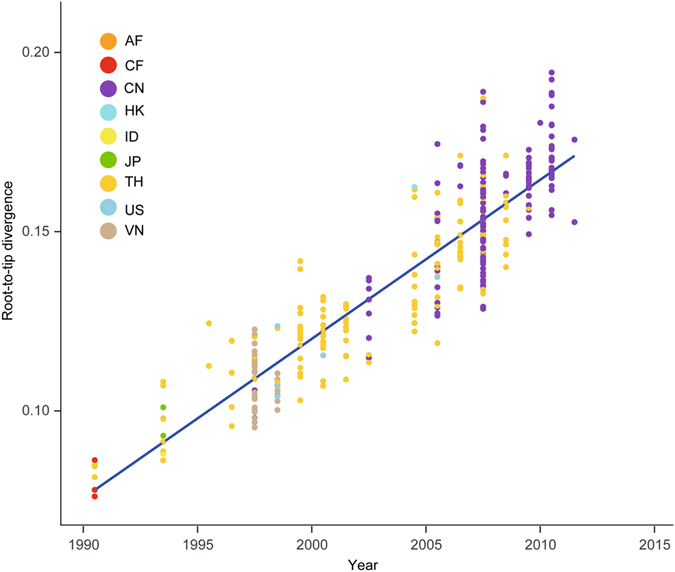
Regression of the root-to-tip genetic distance against year of sampling for 334 HIV-1 CRF01_AE sequences. Genetic distances are based on the tree in Supplementary Figure S2. Colors indicate different sampling locations.

A plot of root-to-tip genetic distance against year of sampling indicated a strong temporal signal with no clear outlier sequences (correlation coefficient = 0.91; slope = 4.74 × 10^−3^), reflecting a relatively clocklike pattern of molecular evolution (Fig. 3). The estimated evolutionary rate for the data set using least-squares dating was 4.60 × 10^−3^ substitutions per site per year. In our Bayesian phylogenetic analysis, we estimated a substitution rate of 4.70 × 10^−3^ substitutions per site per year (95% credibility interval: 4.46 × 10^−3^–4.92 × 10^−3^). The rate estimates from the three methods are in close agreement, as expected when there is low rate variation across branches and a low degree of age clustering among the tips^46^.

The age of each CRF01_AE cluster was also estimated in the analysis (Supplementary Table S2). The first divergences between the sequences from Central African Republic and Thailand were estimated to have occurred in 1974 (95% credibility interval: 1972–1977) and 1981 (95% credibility interval: 1980–1983), respectively. These estimates are consistent with those obtained by Feng *et al*.^18^.

**Figure 4:**
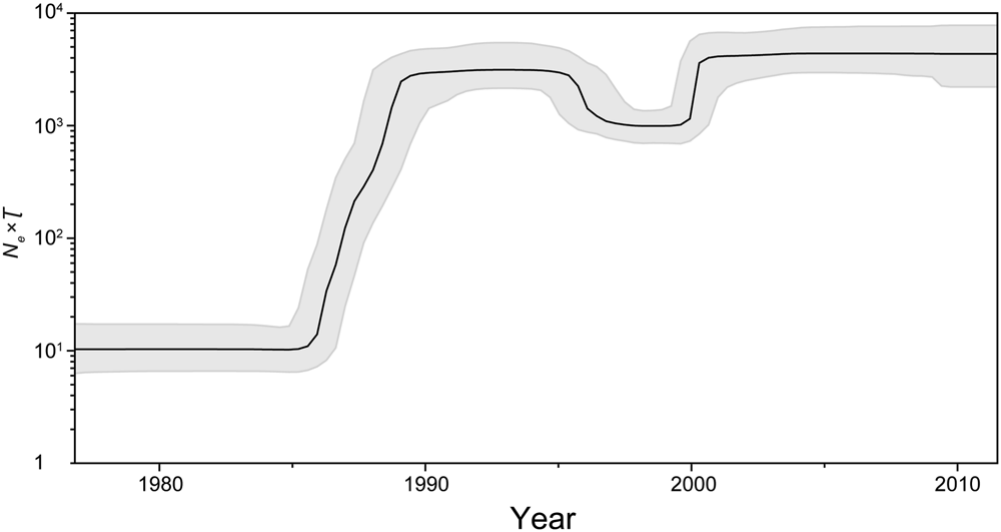
Bayesian skyline demographic reconstruction of HIV-1 CRF01_AE. The vertical axis shows the effective number of infections (*N*_*e*_) multiplied by mean viral generation time (τ). The solid line and shaded region represent the median and 95% credibility interval of *N*_*e*_τ through time.

**Figure 5:**
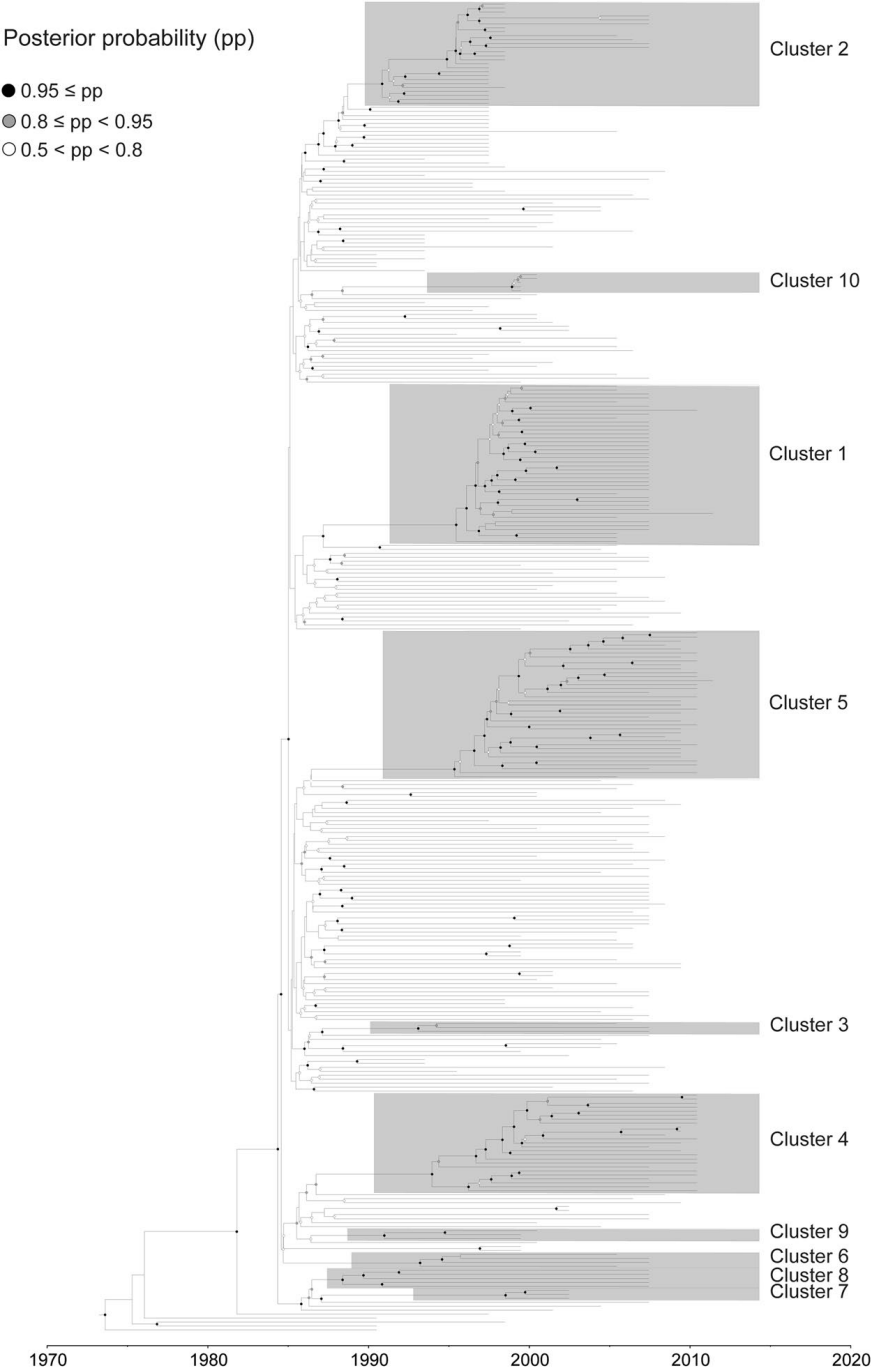
Maximum-clade-credibility tree estimated from near-complete genome sequences of HIV-1 CRF01_AE. Sequence names include accession number, geographic location, and year of sampling. Only internal nodes with posterior probability support >0.5 are shown with white, grey, and black circles.

We further investigated the past population dynamics of CRF01_AE using a Bayesian skyline plot, which depicts the changes in effective population size over time^43^. The effective population size seems to have experienced a complex dynamic, characterized by two phases of exponential growth (1985–1988 and 1999–2000) separated by a periods of constant or declining population size (Fig. 4). The estimates of the phylogenetic relationships among the CRF01_AE sequences using Bayesian coalescent framework were consistent with those inferred using maximum likelihood (Fig. 5).

### Phylogenetic association with geographic location and risk group

Based on the AI and PS statistics, we rejected the null hypothesis of no association between the selected trait (geographic location or risk group) and the phylogeny (*P* < 0.001; Tables 4 and 5). For the MC statistic, we also rejected the null hypothesis of no association between geographic location and the structure of the phylogeny (P < 0.05), with the exception of the MC (ID), MC (HK), and MC (AF) statistics because of insufficient sample sizes from these geographic locations (n = 1; Table 4). For the risk group, the MC (Hetero), MC (MSM), and MC (n/a) statistics rejected the null hypothesis of no association with the structure of the phylogeny (*P* < 0.05; Table 5). However, the MC (IDU) statistic was not significantly larger than expected by chance (*P* = 0.192), and the MC (MTCT) and MC (ST) statistics were not different from those expected by chance. The results of our detailed analysis of geographic location are summarized in Supplementary Tables S3, S4, and S5.

**Table 4:** Statistical analysis of geographic location of CRF01_AE sequences.

Statistic	No. of sequences	Observed mean (95% CI)	Null mean (95% CI)	*P*-value
AI		10.9 (10.0, 11.8)	23.9 (21.7, 26.1)	<0.001*
PS		82.5 (80.0, 85.0)	139.7 (132.3, 147.1)	<0.001*
MC (AF)	1	1.0 (1.0, 1.0)	1.0 (1.0, 1.0)	N/A
MC (CF)	3	1.8 (1.0, 2.0)	1.0 (1.0, 1.0)	0.005*
MC (CN)	154	10.0 (10.0, 10.0)	4.5 (3.1, 6.1)	0.001*
MC (HK)	1	1.0 (1.0, 1.0)	1.0 (1.0, 1.0)	N/A
MC (ID)	1	1.0 (1.0, 1.0)	1.0 (1.0, 1.0)	N/A
MC (JP)	2	2.0 (2.0, 2.0)	1.0 (1.0, 1.0)	0.002*
MC (TH)	134	40.0 (40.0, 40.0)	3.9 (3.0, 5.5)	0.001*
MC (US)	5	2.0 (2.0, 2.0)	1.0 (1.0, 1.0)	0.008*
MC (VN)	33	5.0 (5.0, 5.0)	1.7 (1.0, 2.3)	0.001*

AI, association index.

PS, parsimony score.

MC, monophyletic clade statistic.

95% CI, 95% credbility interval.

*Statistically significant (P < 0.05).

N/A, not available because of the observed 95% CI contains the null 95% CI.

**Table 5:** Statistical analysis of risk group for CRF01_AE sequences.

Statistic	No. of sequences	Observed mean (95% CI)	Null mean (95% CI)	*P*-value
AI		20.1 (19.2, 21.1)	29.7 (27.7, 31.7)	<0.001*
PS		133.2 (130.0, 136.0)	184.4 (176.8, 191.7)	<0.001*
MC (Hetero)	56	5.0 (5.0, 5.0)	3.0 (2.0, 4.1)	0.017*
MC (IDU)	21	3.0 (3.0, 3.0)	2.4 (2.0, 3.1)	0.192
MC (MSM)	56	5.0 (5.0, 5.0)	2.2 (1.8, 3.0)	0.002*
MC (MTCT)	3	1.0 (1.0, 1.0)	1.0 (1.0, 1.0)	N/A
MC (ST)	9	1.0 (1.0, 1.0)	1.0 (1.0, 1.0)	N/A
MC (n/a)	9	40.0 (40.0, 40.0)	3.1 (2.1, 4.5)	0.001*

AI, association index.

PS, parsimony score.

MC, monophyletic clade statistic.

95% CI, 95% credibility interval.

*Statistically significant (P < 0.05).

Hetero, heterosexual.

IDU, injecting drug user.

MSM, men who have sex with men.

MTCT, mother-to-child transmission.

ST, sexual transmission, unspecified type.

n/a, not available.

N/A, not available because of the observed 95% CI contains the null 95% CI.

## Discussion

### Phylodynamics of HIV-1 CRF01_AE

The most prevalent genetic type of HIV-1 in Asia is CRF01_AE. Our evolutionary analyses, based on all of the available near-complete genome sequences of CRF01_AE that included country of origin and year of sampling, revealed the presence of 10 independent clusters. These strongly supported clusters represent founder variants that led to substantial viral spread, most likely into a highly active, high-risk group of HIV-naïve individuals. Population-level transmission depends on the probabilities of transmission and the structure of the social/sexual networks into which a founder virus enters^47–50^. Subsequent transmissions of a founder virus might remain limited to its original transmission network, but can eventually move outside the network and spread regionally, nationally, or even globally.

The basal divergences within HIV-1 CRF01_AE involved the samples not only from Hetero patients collected from the Central African Republic in 1990, but also from a Hetero patient in US collected in 1998. It was previously proposed that HIV-1 CRF01_AE outbreaks in Thailand were directly seeded by the HIV-1 CRF01_AE strains of African origin^9, 12, 13^. We identified 10 independent clusters within the CRF01_AE pandemic, including eight detected in China, and the origins of these clusters date from the late 1980s to the late 1990s. The sequences within some of the clusters were quite dispersed and were identified in as many as eight Chinese provinces. These results support a scenario of multiple CRF01_AE founder viruses that were introduced into epidemiologically linked, high-risk groups in China. As these founder viruses spread within transmission/social/sexual networks, they became the ancestors of each of these independent clusters. Further sampling might reveal the presence of additional HIV-1 CRF01_AE clusters. As more sequences are characterized within other countries, more local, regional, national or global clusters are likely to emerge, presenting a challenge to HIV nomenclature.

Our Bayesian skyline plot analysis revealed that a bottleneck occurred in the second half of the 1990s. This is consistent with demographic data on the decline of HIV prevalence among female commercial sex workers and male sexually transmitted disease patients in Thailand during this period (Supplementary Figure S5). This was most likely a result of the implementation of effective HIV-control measures in Thailand beginning in the late 1980s, including the 100% Condom Program^51–53^. The second period of population growth around 2000 coincided with China’s first explosive travel to Thailand during the late 1990s to early 2000s (Supplementary Table S6)^54^. Therefore, it is tempting to speculate that China’s “free travel” policy provided an opportunity for the establishment, spatial dissemination, and epidemic growth of multiple clusters of CRF01_AE strains from Thailand to China. Furthermore, we found that geographic locations and risk groups are indeed having a significant influence on the complex transmission dynamics of CRF01_AE. The phylogeny of CRF01_AE is likely to have been structured by geography and risk-group traits, especially for China, Thailand, and Vietnam, and for those in the MSM risk group.

### HIV-1 nomenclature

The official nomenclature of HIV-1 includes groups M, N, O, and P, each of which represents a single zoonotic transmission event. Subtypes within the HIV-1 M group were formed by epidemiological factors. The circulating recombinant forms include viruses such as CRF01_AE, CRF02_AG, and CRF04_cpx, which are recombinant viruses with some parts of their genomes clustering with more than one subtype. The official nomenclature also includes some “sub-subtypes” such as A1, A2, F1, and F2, each of which is nearly as distant from each other as subtype B is from subtype D.

There have also been many unofficial designations for local strains and subclades within subtypes, such as the “B-prime” or “Thai-B” and “A3” and “A4” viruses. Identifying subclades within a subtype without standards to name them can lead to a great deal of confusion. For example, Feng *et al*.^18^ reported seven distinct phylogenetic clusters of CRF01_AE strains from China. However, their “Cluster 2” was the same as “Cluster 3” first identified among IDUs in northern Vietnam and the nearby Chinese province of Guangxi^17^, and was also called “IMC-1” by Shiino *et al*.^55^. The best way to standardize the nomenclature of these new phylogenetic clusters is to provide well-characterized reference sequences and to employ the same cluster-identification strategies.

We propose that the 10 HIV-1 CRF01_AE clusters be designated as CRF01_1AE through CRF01_10AE. These clusters are labeled with numbers rather than letters placed before the suffix “AE”. We are proposing that as new clusters AE are identified, reference sequences should be made available in a public HIV database and that the authors use the next available number. Therefore, we are suggesting a method by which HIV-1 CRF01_AE cluster nomenclature will provide a consistent and standardized method to name newly identified transmission-derived clusters among all subtypes and CRFs. We are also providing well-characterized near-complete genome sequences of CRF01_AE as reference sequences by selecting the sequence that had the deepest branch in each of the 10 currently identified CRF01_AE clusters (Supplementary Table S7).

New CRF01_AE cluster reference sequences should include the nomenclature, the representative sequence name, the accession number, the year of sampling, the country of sampling (origin), associated publication(s), and any other demographic information. The more HIV samples that are sequenced and characterized within countries and regions, the more unique clusters that are likely to be identified. This will present challenges to nomenclature and our ability to refer to these variants in a consistent and standardized way.

## Electronic supplementary material

Supplementary Information
