# Supplementary material for: Tracing the epidemic history of HIV-1 CRF01_AE clusters using near-complete genome sequences
Source: Sci Rep. 2017 Jun 22;7:4024. doi: 10.1038/s41598-017-03820-8 (PMC5481428; doi:10.1038/s41598-017-03820-8)
Supplement: Supplementary file 1 — Supplementary Information [file 41598_2017_3820_MOESM1_ESM.pdf]

Tracing the epidemic history of HIV-1 CRF01_AE clusters using near-complete genome sequences

Xingguang LI[1, 2, #], Haizhou LIU[3, #], Lu LIU[4, 5, #], Yi FENG[1, 2], Marcia L. KALISH[6], Simon Y. W. HO[7] and Yiming SHAO[1, 2]

1. State Key Laboratory for Infectious Disease Prevention and Control, National Center for AIDS/STD Control and Prevention, Chinese Center for Disease Control and Prevention, Beijing, China.
2. Collaborative Innovation Center for Diagnosis and Treatment of Infectious Diseases, Hangzhou, Zhejiang, China.
3. Centre for Emerging Infectious Diseases, The State Key Laboratory of Virology, Wuhan Institute of Virology, University of Chinese Academy of Sciences, Wuhan, China.
4. Shantou University Medical College, Shantou 515041, China.
5. College of Veterinary Medicine, South China Agricultural University, Guangzhou 510642, China.
6. Vanderbilt Institute for Global Health, Vanderbilt University School of Medicine, Nashville, Tennessee, USA.
7. School of Life and Environmental Sciences, University of Sydney, Sydney, New

South Wales 2006, Australia.

Corresponding author:

Yiming SHAO, Division of Research on Virology and Immunology, National Center for AIDS/STD Control and Prevention, Chinese Center for Disease Control and Prevention, No. 155 Changbai Road, Changping District, Beijing 102206, China. Tel: +86 10 58900981; Fax: +86 10 58900980; E-mail: yshao08@gmail.com.

Condensed Title: The epidemic history of HIV-1 CRF01_AE clusters

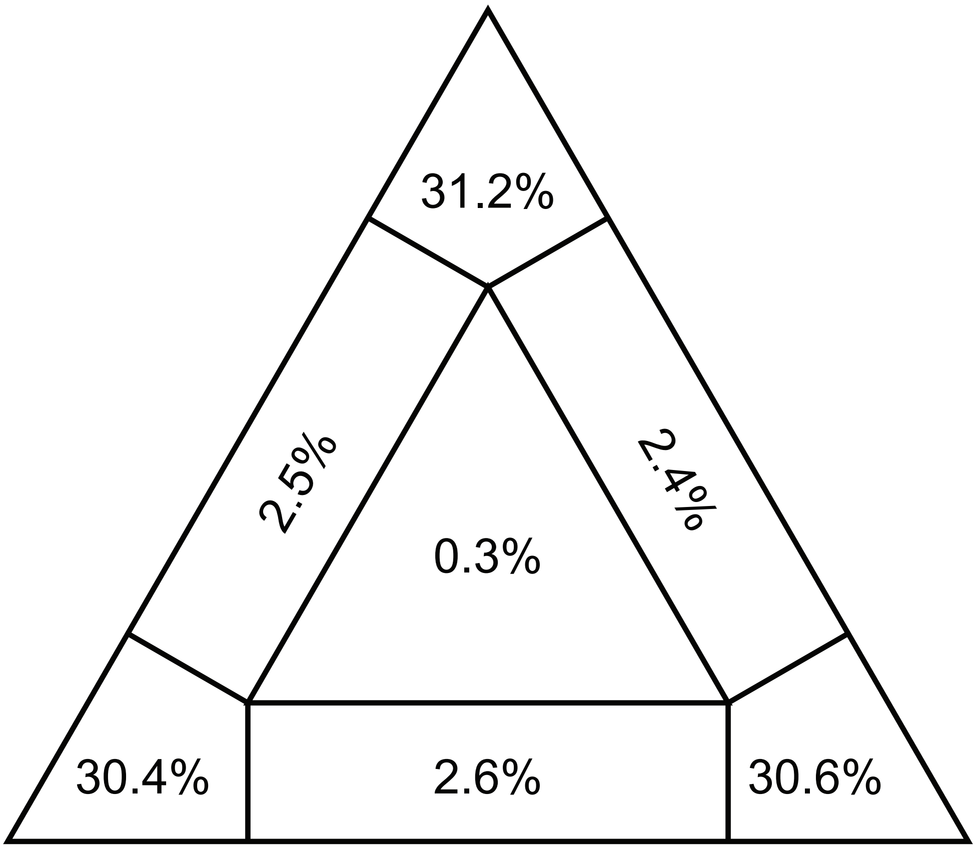

Supplementary Figure S1. Likelihood-mapping analyses of near-complete genome sequences of HIV-1 CRF01_AE. The likelihoods of the three tree topologies of each possible quartet (or of a random sample of quartets) are denoted by a data point in an equilateral triangle. The distribution of points in the seven areas of the triangle reflects the tree-likeness of the data. Specifically, the three corners represent fully resolved tree topologies; the center represents an unresolved (star) phylogeny; and the sides represent support for conflicting tree topologies.

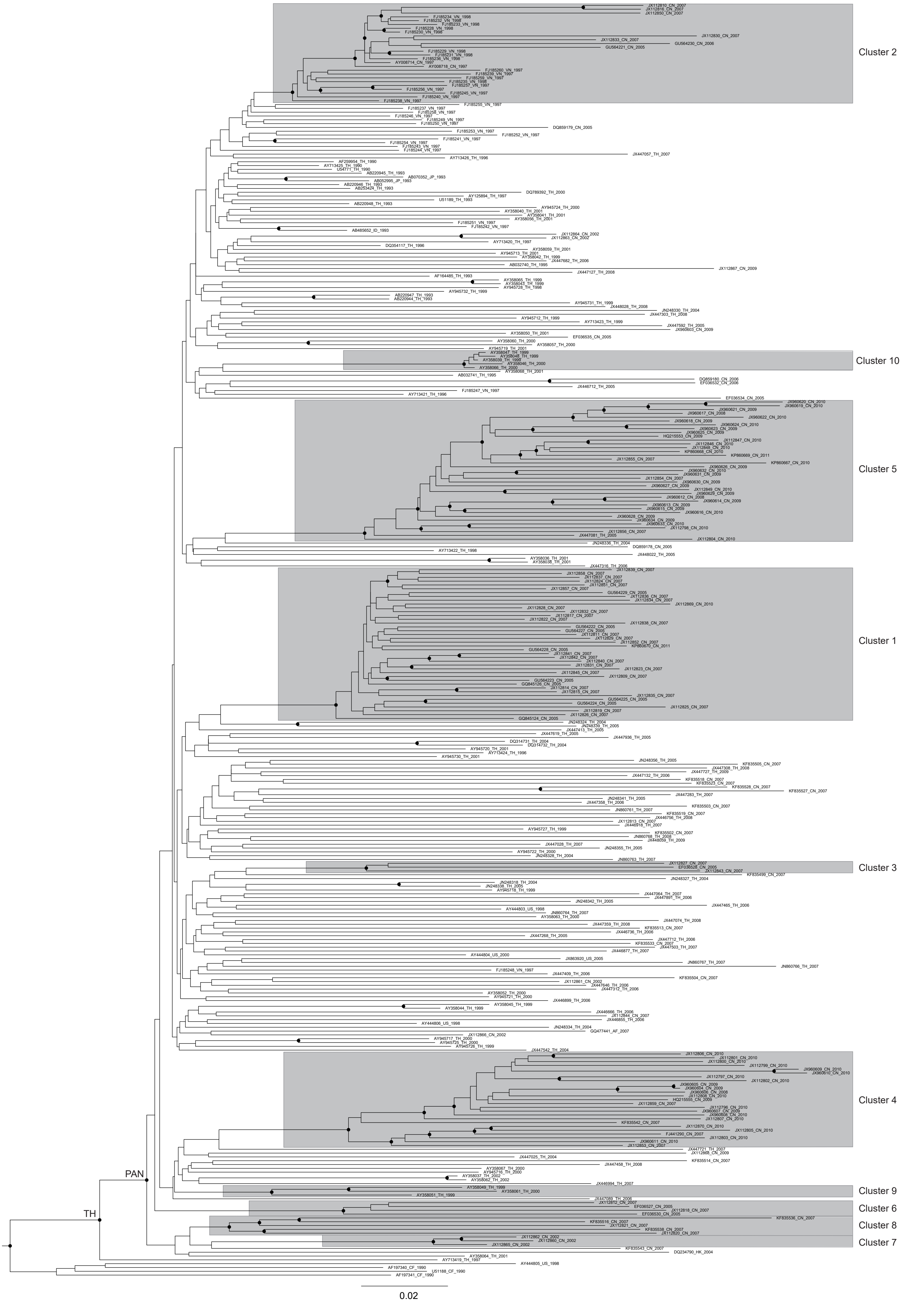

Supplementary Figure S2. Phylogenetic relationships of near-complete genome sequences from HIV-1 CRF01_AE. The tree was inferred using a maximum-likelihood analysis of 334 near-complete genome sequences of HIV-1 CRF01_AE plus four reference sequences of HIV-1 subtype B as outgroup. For visual clarity, only HIV-1 CRF01_AE strains are shown. Bootstrap value ≥90% is shown at the node with black circle. Sequence names include accession number, geographic location, and year of sampling. The scale bar shows nucleotide substitutions per site. Shading indicates the delineation of 10 CRF01_AE clusters. TH = Thailand HIV-1 CRF01_AE; PAN = Pandemic HIV-1 CRF01_AE.

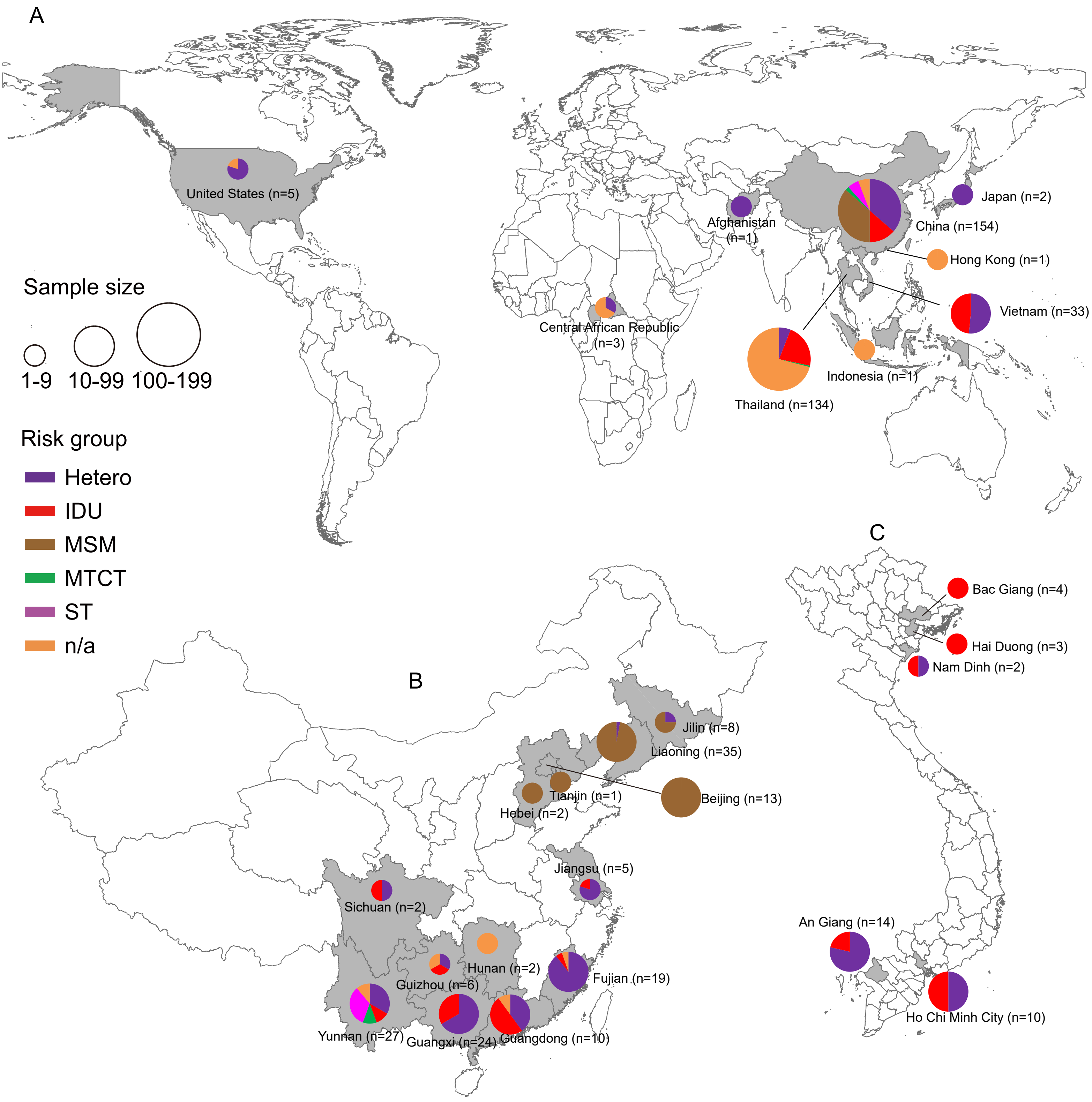

Supplementary Figure S3. Geographic distribution of risk groups of HIV-1 CRF01_AE identified in the present study. Geographic distribution of risk groups of HIV-1 CRF01_AE is shown at the (A) country level, and at the provincial level for (B) China and (C) Vietnam. Each risk group of HIV-1 CRF01_AE identified in this study is color-coded, as shown on the left. The figure was created using Adobe Illustrator CS5 version 15.0.0 software, based on the maps obtained from Craft MAP website (http://www.craftmap.box-i.net/).

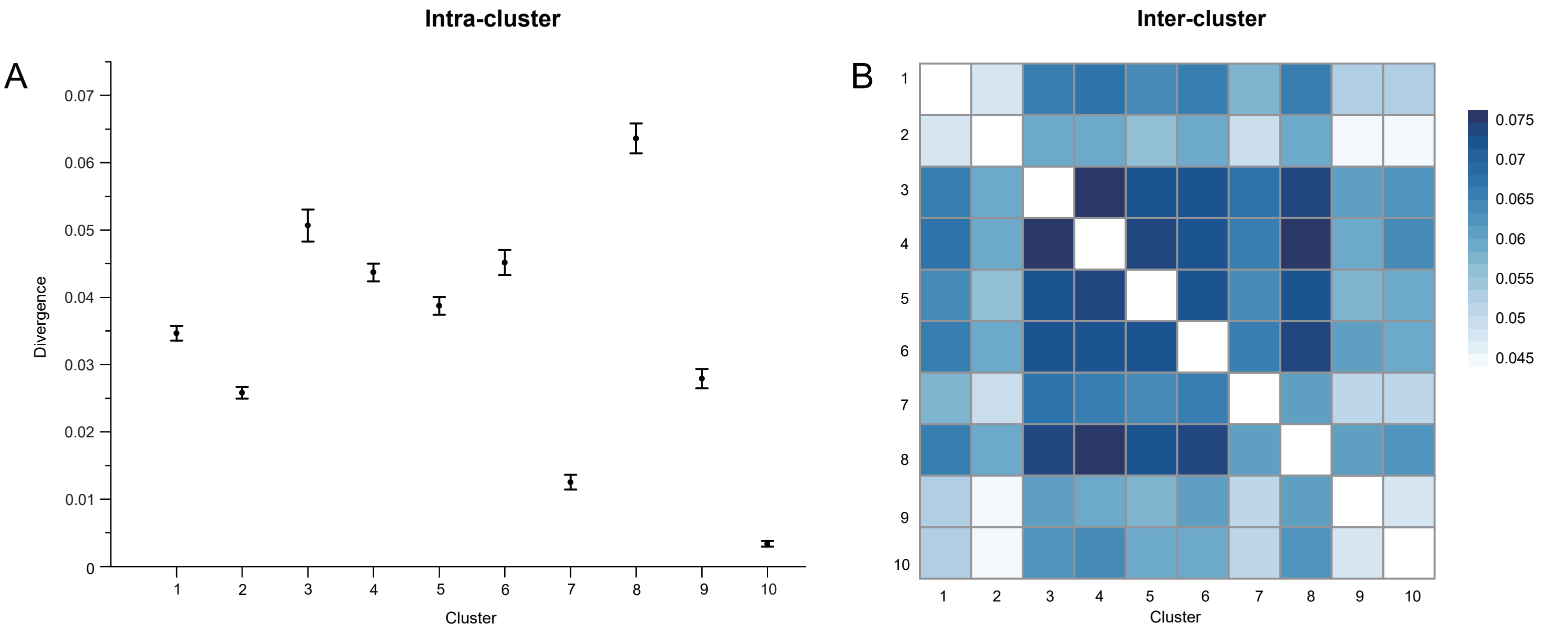

Supplementary Figure S4. Average pairwise genetic distances within and between HIV-1 CRF01_AE clusters. The number of base substitutions per site, averaged across all sequence pairs within (A) and between (B) HIV-1 CRF01_AE clusters.

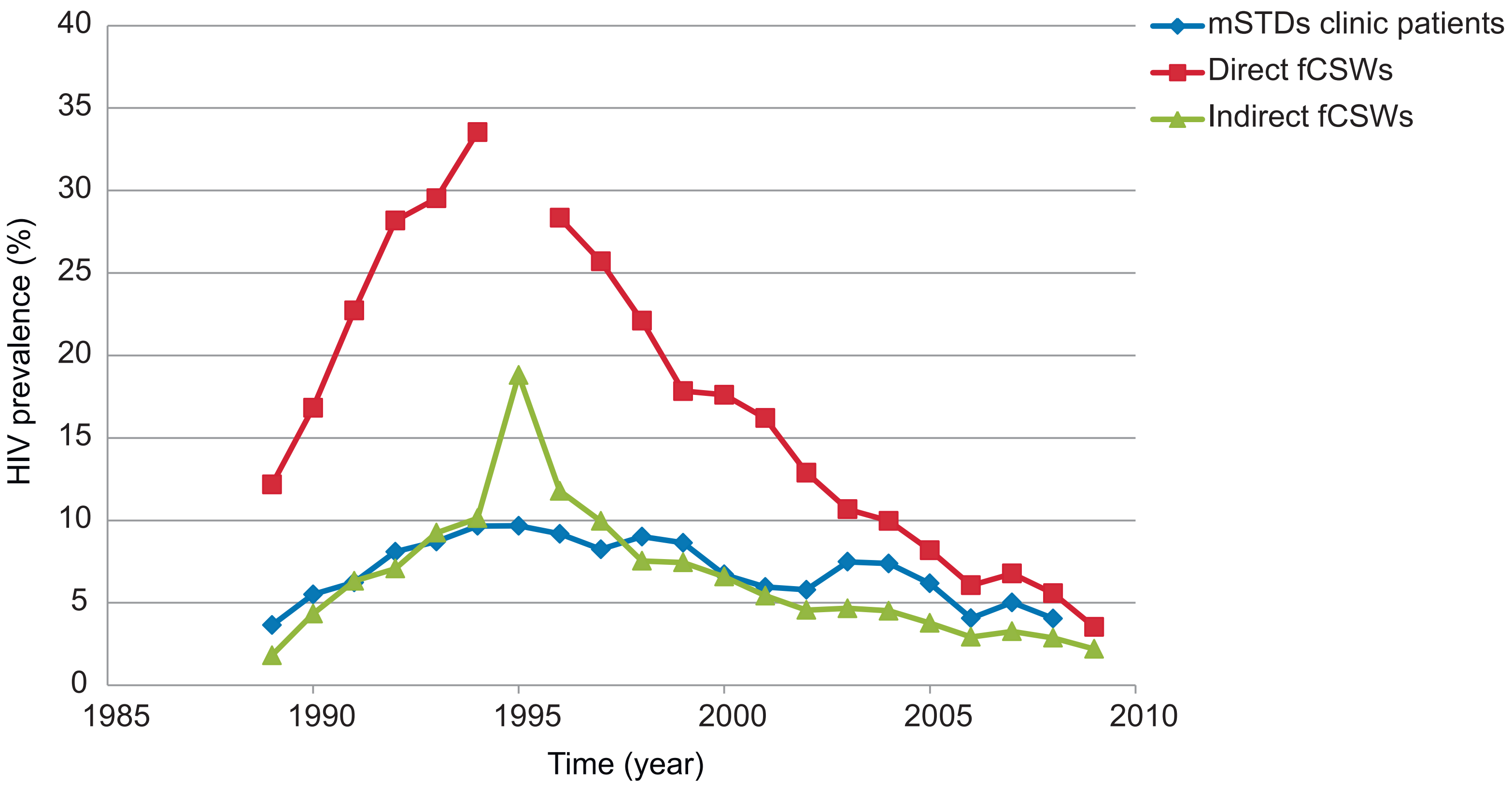

Supplementary Figure S5. HIV prevalence among female commercial sex workers (fCSWs) and male sexually transmitted disease patients (mSTDs) in Thailand by year. HIV prevalence is given for brothel-based female commercial sex workers (direct fCSWs) and non-brothel-based female commercial sex workers (indirect fCSWs). Data were unavailable for direct fCSWs in Thailand in 1995. Data were obtained from the Bureau of Epidemiology, Thailand.

Supplementary Table S1. HIV-1 CRF01_AE sequences analysed in the present study.

Acc. No.	Sequence name	Geographic source	Sampling year	Risk factor[a]	CRF01_AE cluster	Data source
GQ845124	05GX034	Guangxi, China	2005	Hetero	CRF01_1AE	database
JX112835	GX070143	Guangxi, China	2007	Hetero	CRF01_1AE	database
JX112832	GX070043	Guangxi, China	2007	Hetero	CRF01_1AE	database
JX112834	GX070076	Guangxi, China	2007	Hetero	CRF01_1AE	database
GU564229	05GX162	Guangxi, China	2005	Hetero	CRF01_1AE	database
JX112836	GX070145	Guangxi, China	2007	Hetero	CRF01_1AE	database
GU564224	05GX013	Guangxi, China	2005	IDU	CRF01_1AE	database
GU564225	05GX014	Guangxi, China	2005	Hetero	CRF01_1AE	database
JX112837	GX070149	Guangxi, China	2007	IDU	CRF01_1AE	database
JX112838	GX070154	Guangxi, China	2007	IDU	CRF01_1AE	database
JX112839	GX070167	Guangxi, China	2007	IDU	CRF01_1AE	database
JX112829	GX070003	Guangxi, China	2007	Hetero	CRF01_1AE	database
GQ845126	05GX128	Guangxi, China	2005	Hetero	CRF01_1AE	database
GU564227	05GX142	Guangxi, China	2005	Hetero	CRF01_1AE	database
GU564222	05GX002	Guangxi, China	2005	Hetero	CRF01_1AE	database
GU564228	05GX156	Guangxi, China	2005	IDU	CRF01_1AE	database
GU564223	05GX012	Guangxi, China	2005	Hetero	CRF01_1AE	database
JX112831	GX070006	Guangxi, China	2007	IDU	CRF01_1AE	database
JX112819	GD070010	Guangdong, China	2007	Hetero	CRF01_1AE	database
JX112825	GD070096	Guangdong, China	2007	IDU	CRF01_1AE	database
JX112826	GD070118	Guangdong, China	2007	IDU	CRF01_1AE	database
JX112828	GD070176	Guangdong, China	2007	IDU	CRF01_1AE	database
JX112822	GD070083	Guangdong, China	2007	Hetero	CRF01_1AE	database
JX112823	GD070090	Guangdong, China	2007	IDU	CRF01_1AE	database
JX112824	GD070092	Guangdong, China	2007	IDU	CRF01_1AE	database
JX112809	FJ070010	Fujian, China	2007	Hetero	CRF01_1AE	database
JX112817	FJ070040	Fujian, China	2007	Hetero	CRF01_1AE	database
JX112814	FJ070035	Fujian, China	2007	Hetero	CRF01_1AE	database
JX112815	FJ070037	Fujian, China	2007	Hetero	CRF01_1AE	database
JX112811	FJ070017	Fujian, China	2007	Hetero	CRF01_1AE	database
JX112845	GZ070127	Guizhou,China	2007	Hetero	CRF01_1AE	database
JX112840	GZ070004	Guizhou, China	2007	IDU	CRF01_1AE	database
JX112841	GZ070015	Guizhou,China	2007	n/a	CRF01_1AE	database
JX112842	GZ070016	Guizhou,China	2007	IDU	CRF01_1AE	database
JX112851	JS071001	Jiangsu, China	2007	Hetero	CRF01_1AE	database
JX112852	JS071004	Jiangsu, China	2007	Hetero	CRF01_1AE	database
JX112857	SC070062	Sichuan, China	2007	IDU	CRF01_1AE	database
JX112858	SC070064	Sichuan, China	2007	Hetero	CRF01_1AE	database
JX112869	ZK052	Hunan, China	2010	n/a	CRF01_1AE	database
KP860670	JL110056	Jilin, China	2011	Hetero	CRF01_1AE	this study
AY008714	97CNGX2F	Guangxi, China	1997	IDU	CRF01_2AE	database
AY008718	97CNGX11F	Guangxi, China	1997	IDU	CRF01_2AE	database
GU564221	05GX001	Guangxi, China	2005	Hetero	CRF01_2AE	database
GU564230	06GX239	Guangxi, China	2006	Hetero	CRF01_2AE	database
JX112830	GX070005	Guangxi, China	2007	Hetero	CRF01_2AE	database
JX112833	GX070044	Guangxi, China	2007	Hetero	CRF01_2AE	database
JX112816	FJ070039	Fujian, China	2007	Hetero	CRF01_2AE	database
JX112810	FJ070013	Fujian, China	2007	Hetero	CRF01_2AE	database
JX112850	JS070901	Jiangsu, China	2007	Hetero	CRF01_2AE	database
FJ185238	97VNHCM302	Hochiminh, Vietnam	1997	IDU	CRF01_2AE	database
FJ185239	97VNHCM303	Hochiminh, Vietnam	1997	IDU	CRF01_2AE	database

FJ185259	97VNHCM306	Hochiminh, Vietnam	1997	IDU	CRF01_2AE	database
FJ185260	97VNHCM309	Hochiminh, Vietnam	1997	IDU	CRF01_2AE	database
FJ185240	97VNHCM310	Hochiminh, Vietnam	1997	IDU	CRF01_2AE	database
FJ185228	98VNBG4	Bacgiang, Vietnam	1998	IDU	CRF01_2AE	database
FJ185229	98VNBG5	Bacgiang, Vietnam	1998	IDU	CRF01_2AE	database
FJ185230	98VNBG6	Bacgiang, Vietnam	1998	IDU	CRF01_2AE	database
FJ185231	98VNBG7	Bacgiang, Vietnam	1998	IDU	CRF01_2AE	database
FJ185245	97VNAG201	Angiang, Vietnam	1997	IDU	CRF01_2AE	database
FJ185256	97VNAG220	Angiang, Vietnam	1997	IDU	CRF01_2AE	database
FJ185257	97VNAG221	Angiang, Vietnam	1997	IDU	CRF01_2AE	database
FJ185232	98VNHD9	Haiduong, Vietnam	1998	IDU	CRF01_2AE	database
FJ185233	98VNHD10	Haiduong, Vietnam	1998	IDU	CRF01_2AE	database
FJ185234	98VNHD11	Haiduong, Vietnam	1998	IDU	CRF01_2AE	database
FJ185235	98VNND15	Namdinh, Vietnam	1998	Hetero	CRF01_2AE	database
FJ185236	98VNND17	Namdinh, Vietnam	1998	IDU	CRF01_2AE	database
EF036528	FJ052	Fujian, China	2005	IDU	CRF01_3AE	database
JX112827	GD070120	Guangdong, China	2007	n/a	CRF01_3AE	database
JX112843	GZ070123	Guizhou, China	2007	Hetero	CRF01_3AE	database
JX112796	CYM059	Beijing, China	2010	MSM	CRF01_4AE	database
JX112797	CYM075	Beijing, China	2010	MSM	CRF01_4AE	database
JX112799	CYM124	Beijing, China	2010	MSM	CRF01_4AE	database
JX112800	CYM136	Beijing, China	2010	MSM	CRF01_4AE	database
JX112801	CYM138	Beijing, China	2010	MSM	CRF01_4AE	database
JX112802	CYM139	Beijing, China	2010	MSM	CRF01_4AE	database
JX112806	CYM149	Beijing, China	2010	MSM	CRF01_4AE	database
JX112807	CYM152	Beijing, China	2010	MSM	CRF01_4AE	database
JX112808	CYM154	Beijing, China	2010	MSM	CRF01_4AE	database
JX112803	CYM140	Beijing, China	2010	MSM	CRF01_4AE	database
JX112805	CYM147	Beijing, China	2010	MSM	CRF01_4AE	database
JX960611	10LNA571	Liaoning, China	2010	MSM	CRF01_4AE	database
JX960609	10LNA124	Liaoning, China	2010	MSM	CRF01_4AE	database
JX960610	10LNA819	Liaoning, China	2010	MSM	CRF01_4AE	database
JX960604	09LNA008	Liaoning, China	2009	MSM	CRF01_4AE	database
JX960605	09LNA011	Liaoning, China	2009	MSM	CRF01_4AE	database
JX960606	08LNA003	Liaoning, China	2008	MSM	CRF01_4AE	database
JX960607	09LNA340	Liaoning, China	2009	MSM	CRF01_4AE	database
JX960608	10LNA103	Liaoning, China	2010	MSM	CRF01_4AE	database
FJ441290	07JSWX045	Jiangsu, China	2007	IDU	CRF01_4AE	database
JX112853	JS071101	Jiangsu, China	2007	Hetero	CRF01_4AE	database
JX112870	ZK056	Hunan, China	2010	n/a	CRF01_4AE	database
HQ215555	1109	Hebei, China	2009	MSM	CRF01_4AE	database
JX112859	TJ070003	Tianjin, China	2007	MSM	CRF01_4AE	database
KF835542	07CNYN364	Yunnan, China	2007	ST	CRF01_4AE	database
JX960612	08LNA002	Liaoning, China	2008	MSM	CRF01_5AE	database
JX960614	09LNA230	Liaoning, China	2009	MSM	CRF01_5AE	database
JX960613	09LNA020	Liaoning, China	2009	MSM	CRF01_5AE	database
JX960615	09LNA040	Liaoning, China	2009	MSM	CRF01_5AE	database
JX960616	10LNA821	Liaoning, China	2010	MSM	CRF01_5AE	database
JX960617	08LNA004	Liaoning, China	2008	MSM	CRF01_5AE	database
JX960618	09LNA041	Liaoning, China	2009	MSM	CRF01_5AE	database
JX960619	10LNA294	Liaoning, China	2010	MSM	CRF01_5AE	database
JX960620	10LNA918	Liaoning, China	2010	MSM	CRF01_5AE	database
JX960621	09LNA425	Liaoning, China	2009	MSM	CRF01_5AE	database
JX960622	10LNA264	Liaoning, China	2010	MSM	CRF01_5AE	database
JX960623	09LNA013	Liaoning, China	2009	MSM	CRF01_5AE	database
JX960624	10LNA669	Liaoning, China	2010	MSM	CRF01_5AE	database

JX960625	09LNA527	Liaoning, China	2009	MSM	CRF01_5AE	database
JX960626	10LNA105	Liaoning, China	2009	MSM	CRF01_5AE	database
JX960627	09LNA007	Liaoning, China	2009	MSM	CRF01_5AE	database
JX960628	09LNA353	Liaoning, China	2009	MSM	CRF01_5AE	database
JX960629	10LNA016	Liaoning, China	2009	MSM	CRF01_5AE	database
JX960630	09LNA005	Liaoning, China	2009	MSM	CRF01_5AE	database
JX960631	09LNA136	Liaoning, China	2009	MSM	CRF01_5AE	database
JX960632	10LNA976	Liaoning, China	2010	MSM	CRF01_5AE	database
JX960633	10LNA057	Liaoning, China	2010	MSM	CRF01_5AE	database
JX960634	09LNA025	Liaoning, China	2009	MSM	CRF01_5AE	database
JX112854	LN070008	Liaoning, China	2007	MSM	CRF01_5AE	database
JX112855	LN070010	Liaoning, China	2007	MSM	CRF01_5AE	database
JX112856	LN070013	Liaoning, China	2007	Hetero	CRF01_5AE	database
JX112846	JL100005	Jilin, China	2010	Hetero	CRF01_5AE	database
JX112847	JL100007	Jilin, China	2010	MSM	CRF01_5AE	database
JX112848	JL100014	Jilin, China	2010	MSM	CRF01_5AE	database
JX112849	JL100020	Jilin, China	2010	MSM	CRF01_5AE	database
KP860667	JL100034	Jilin, China	2010	MSM	CRF01_5AE	this study
KP860668	JL100038	Jilin, China	2010	MSM	CRF01_5AE	this study
KP860669	JL110010	Jilin, China	2011	MSM	CRF01_5AE	this study
JX112798	CYM105	Beijing, China	2010	MSM	CRF01_5AE	database
JX112804	CMY143	Beijing, China	2010	MSM	CRF01_5AE	database
HQ215553	1119	Hebei, China	2009	MSM	CRF01_5AE	database
JX447081	AA033a_wg6a	Thailand	2005	n/a	CRF01_5AE	database
EF036527	FJ055	Fujian, China	2005	Hetero	CRF01_6AE	database
EF036530	FJ057	Fujian, China	2005	Hetero	CRF01_6AE	database
JX112812	FJ070018	Fujian, China	2007	Hetero	CRF01_6AE	database
JX112818	FJ070043	Fujian, China	2007	Hetero	CRF01_6AE	database
JX112860	YN0203	Yunnan, China	2002	Hetero	CRF01_7AE	database
JX112862	YN0225	Yunnan, China	2002	Hetero	CRF01_7AE	database
JX112865	YN0235	Yunnan, China	2002	Hetero	CRF01_7AE	database
KF835538	07CNYN359	Yunnan, China	2007	Hetero	CRF01_8AE	database
KF835516	07CNYN329	Yunnan, China	2007	n/a	CRF01_8AE	database
KF835536	07CNYN357	Yunnan, China	2007	ST	CRF01_8AE	database
JX112820	GD070058	Guangdong, China	2007	Hetero	CRF01_8AE	database
JX112821	GD070059	Guangdong, China	2007	Hetero	CRF01_8AE	database
AY358049	OUR258I	Thailand	1999	IDU	CRF01_9AE	database
AY358061	OUR746I	Thailand	2000	IDU	CRF01_9AE	database
AY358051	OUR422I	Thailand	1999	IDU	CRF01_9AE	database
AY358039	OUR199I	Thailand	1999	IDU	CRF01_10AE	database
AY358047	OUR202I	Thailand	1999	IDU	CRF01_10AE	database
AY358048	OUR203I	Thailand	1999	IDU	CRF01_10AE	database
AY358066	OUR200I	Thailand	2000	IDU	CRF01_10AE	database
AY358046	OUR201I	Thailand	2000	IDU	CRF01_10AE	database
JX112867	YN09P0011	Yunnan, China	2009	Hetero		database
JX112868	YN09P0015	Yunnan, China	2009	Hetero		database
JX112861	YN0221	Yunnan, China	2002	IDU		database
JX112863	YN0229	Yunnan, China	2002	IDU		database
JX112864	YN0232	Yunnan, China	2002	Hetero		database
JX112866	YN0236	Yunnan, China	2002	Hetero		database
KF835499	07CNYN312	Yunnan, China	2007	MTCT		database
KF835502	07CNYN315	Yunnan, China	2007	ST		database
KF835503	07CNYN316	Yunnan, China	2007	ST		database
KF835504	07CNYN317	Yunnan, China	2007	n/a		database
KF835505	07CNYN318	Yunnan, China	2007	MTCT		database
KF835513	07CNYN326	Yunnan, China	2007	Hetero		database

KF835514	07CNYN327	Yunnan, China	2007	IDU	database
KF835518	07CNYN332	Yunnan, China	2007	ST	database
KF835519	07CNYN333	Yunnan, China	2007	ST	database
KF835523	07CNYN337	Yunnan, China	2007	MTCT	database
KF835527	07CNYN342	Yunnan, China	2007	ST	database
KF835528	07CNYN343	Yunnan, China	2007	n/a	database
KF835533	07CNYN354	Yunnan, China	2007	ST	database
KF835543	07CNYN366	Yunnan, China	2007	ST	database
DQ859179	FJ053	Fujian, China	2005	Hetero	database
DQ859178	FJ051	Fujian, China	2005	Hetero	database
DQ859180	FJ054	Fujian, China	2006	Hetero	database
EF036532	FJ063	Fujian, China	2006	Hetero	database
EF036534	FJ065	Fujian, China	2005	Hetero	database
EF036535	FJ066	Fujian, China	2005	Hetero	database
JX112813	FJ070033	Fujian, China	2007	n/a	database
JX960603	09LNA379	Liaoning, China	2009	MSM	database
JX112844	GZ070126	Guizhou, China	2007	n/a	database
DQ234790	HK001	Hong Kong	2004	n/a	database
FJ185246	97VNAG202	Angiang, Vietnam	1997	Hetero	database
FJ185252	97VNAG212	Angiang, Vietnam	1997	Hetero	database
FJ185253	97VNAG214	Angiang, Vietnam	1997	Hetero	database
FJ185254	97VNAG216	Angiang, Vietnam	1997	Hetero	database
FJ185255	97VNAG218	Angiang, Vietnam	1997	Hetero	database
FJ185258	97VNAG223	Angiang, Vietnam	1997	Hetero	database
FJ185249	97VNAG207	Angiang, Vietnam	1997	Hetero	database
FJ185250	97VNAG208	Angiang, Vietnam	1997	Hetero	database
FJ185247	97VNAG204	Angiang, Vietnam	1997	Hetero	database
FJ185248	97VNAG206	Angiang, Vietnam	1997	Hetero	database
FJ185251	97VNAG210	Angiang, Vietnam	1997	Hetero	database
FJ185237	97VNHCM301	Hochiminh, Vietnam	1997	Hetero	database
FJ185241	97VNHCM314	Hochiminh, Vietnam	1997	Hetero	database
FJ185242	97VNHCM319	Hochiminh, Vietnam	1997	Hetero	database
FJ185243	97VNHCM343	Hochiminh, Vietnam	1997	Hetero	database
FJ185244	97VNHCM345	Hochiminh, Vietnam	1997	Hetero	database
AY358052	OUR595I	Thailand	2000	IDU	database
AF164485	93TH9021	Thailand	1993	n/a	database
JN248318	04TH107542	Thailand	2004	n/a	database
JN248338	05TH127331	Thailand	2005	n/a	database
AY945718	99TH_C2405	Thailand	1999	n/a	database
AB032740	95TNIH022	Thailand	1995	Hetero	database
AB032741	95TNIH047	Thailand	1995	Hetero	database
AB220944	93TH051	Thailand	1993	n/a	database
AB220945	93TH054	Thailand	1993	n/a	database
AB220946	93TH060	Thailand	1993	n/a	database
AB220947	93TH062	Thailand	1993	n/a	database
AB220948	93TH065	Thailand	1993	IDU	database
AB253424	93TH057	Thailand	1993	IDU	database
AF259954	CM235	Thailand	1990	Hetero	database
AY125894	97TH6_107	Thailand	1997	n/a	database
AY358036	OUR786I	Thailand	2001	IDU	database
AY358037	OUR737I	Thailand	2002	IDU	database
AY358038	OUR674I	Thailand	2001	IDU	database
AY358040	OUR609I	Thailand	2001	IDU	database
AY358041	OUR642I	Thailand	2001	IDU	database
AY358042	OUR044I	Thailand	1999	IDU	database
AY358043	OUR066I	Thailand	1999	IDU	database

AY358044	OUR098I	Thailand	1999	IDU	database
AY358045	OUR164I	Thailand	1999	IDU	database
AY358050	OUR414I	Thailand	2001	Hetero	database
AY358056	OUR647I	Thailand	2001	IDU	database
AY358057	OUR661I	Thailand	2000	IDU	database
AY358059	OUR702I	Thailand	2001	IDU	database
AY358060	OUR724I	Thailand	2000	IDU	database
AY358062	OUR769I	Thailand	2002	IDU	database
AY358063	OUR810I	Thailand	2000	IDU	database
AY358064	OUR830I	Thailand	2001	IDU	database
AY358065	OUR008I	Thailand	1999	IDU	database
AY358067	OUR721I	Thailand	2000	IDU	database
AY358068	OUR788I	Thailand	2001	IDU	database
AY713419	97TH_NP1695	Thailand	1997	n/a	database
AY713420	97TH_NP1525	Thailand	1997	n/a	database
AY713421	96TH_NI1046	Thailand	1996	n/a	database
AY713422	98TH_NP1251	Thailand	1998	n/a	database
AY713423	99TH_NI1052	Thailand	1999	n/a	database
AY713424	96TH_M02138	Thailand	1996	n/a	database
AY713425	90TH_CM244	Thailand	1990	n/a	database
AY945712	99TH_C1080	Thailand	1999	Hetero	database
AY945713	01TH_C1436	Thailand	2001	n/a	database
AY945716	00TH_C2101	Thailand	2000	Hetero	database
AY945717	00TH_C2257	Thailand	2000	n/a	database
AY945719	01TH_C2570	Thailand	2001	n/a	database
AY945720	01TH_C3256	Thailand	2001	n/a	database
AY945721	00TH_C3347	Thailand	2000	n/a	database
AY945722	00TH_C4118	Thailand	2000	n/a	database
AY945724	00TH_C4151	Thailand	2000	n/a	database
AY945725	00TH_C4382	Thailand	2000	n/a	database
AY945726	99TH_C4460	Thailand	1999	n/a	database
AY945727	99TH_R1149	Thailand	1999	Hetero	database
AY945728	98TH_R1166	Thailand	1998	Hetero	database
AY945730	01TH_R2184	Thailand	2001	Hetero	database
AY945731	99TH_R3006	Thailand	1999	Hetero	database
AY945732	99TH_R3265	Thailand	1999	Hetero	database
DQ314731	BKD	Thailand	2004	MTCT	database
DQ314732	BKM	Thailand	2004	n/a	database
DQ354117	M114	Thailand	1996	Hetero	database
DQ789392	C1705	Thailand	2000	n/a	database
JN248324	04TH328531	Thailand	2004	n/a	database
JN248327	04TH427990	Thailand	2004	n/a	database
JN248328	04TH505841	Thailand	2004	n/a	database
JN248330	04TH613543	Thailand	2004	n/a	database
JN248334	04TH807015	Thailand	2004	n/a	database
JN248336	04TH817196	Thailand	2004	n/a	database
JN248339	05TH130087	Thailand	2005	n/a	database
JN248341	05TH327568	Thailand	2005	n/a	database
JN248342	05TH342968	Thailand	2005	n/a	database
JN248355	05TH741452	Thailand	2005	n/a	database
JN248356	05TH841749	Thailand	2005	n/a	database
JN860761	MERLBDTRC2	Thailand	2007	n/a	database
JN860763	MERLBDTRC4	Thailand	2007	n/a	database
JN860764	MERLBDTRC5	Thailand	2007	n/a	database
JN860766	MERLBDTRC7	Thailand	2007	n/a	database
JN860767	MERLBDTRC8	Thailand	2007	n/a	database

JN860768	MERLBDTRC9	Thailand	2008	n/a	database
JX446666	AA002a_WG1	Thailand	2006	n/a	database
JX446712	AA004a_wg4a	Thailand	2005	n/a	database
JX446736	AA006a02	Thailand	2006	n/a	database
JX446756	AA007a_WG10	Thailand	2008	n/a	database
JX446855	AA014a01	Thailand	2006	n/a	database
JX446877	AA015a_WG4	Thailand	2007	n/a	database
JX446899	AA017a_wg1	Thailand	2006	n/a	database
JX446918	AA019a_WG11	Thailand	2007	n/a	database
JX446994	AA024a04	Thailand	2007	n/a	database
JX447025	AA027a_wg1a	Thailand	2004	n/a	database
JX447028	AA028a_wg3	Thailand	2007	n/a	database
JX447057	AA030a04	Thailand	2007	n/a	database
JX447064	AA031a02	Thailand	2007	n/a	database
JX447074	AA032a04	Thailand	2008	n/a	database
JX447089	AA034a_wg2	Thailand	2006	n/a	database
JX447127	AA037a_WG6	Thailand	2008	n/a	database
JX447132	AA038a_WG3	Thailand	2006	n/a	database
JX447268	AA049a_WG13	Thailand	2005	n/a	database
JX447283	AA050a_WG7	Thailand	2007	n/a	database
JX447303	AA052a08	Thailand	2008	n/a	database
JX447308	AA054a06	Thailand	2008	n/a	database
JX447312	AA055a_WG4	Thailand	2006	n/a	database
JX447316	AA056a_WG5	Thailand	2006	n/a	database
JX447358	AA059a_WG9	Thailand	2006	n/a	database
JX447359	AA060a_WG1	Thailand	2008	n/a	database
JX447409	AA063a_WG37	Thailand	2006	n/a	database
JX447413	AA064a_WG2	Thailand	2005	n/a	database
JX447458	AA067a_WG12	Thailand	2008	n/a	database
JX447465	AA068a_14	Thailand	2006	n/a	database
JX447503	AA072a03	Thailand	2007	n/a	database
JX447542	AA075a_WG7	Thailand	2004	n/a	database
JX447592	AA079a_WG4	Thailand	2005	n/a	database
JX447619	AA081a14	Thailand	2005	n/a	database
JX447646	AA082a_WG9	Thailand	2006	n/a	database
JX447682	AA085a_wg2	Thailand	2006	n/a	database
JX447712	AA088a_wg14	Thailand	2006	n/a	database
JX447721	AA089a05	Thailand	2007	n/a	database
JX447727	AA090a_WG11	Thailand	2009	n/a	database
JX447891	AA099a_WG9	Thailand	2006	n/a	database
JX447936	AA101a_WG1	Thailand	2005	n/a	database
JX448022	AA107a_wg4	Thailand	2005	n/a	database
JX448028	AA108a_WG6	Thailand	2008	n/a	database
JX448059	AA111a_WG11	Thailand	2009	n/a	database
U51189	93TH253	Thailand	1993	Hetero	database
AY713426	96TH_NI1149	Thailand	1996	n/a	database
U54771	CM240	Thailand	1990	Hetero	database
AY444803	98US_MSC1120	United States	1998	Hetero	database
AY444806	98US_MSC3012	United States	1998	Hetero	database
JX863920	306163_FL	United States	2005	n/a	database
AY444804	00US_MSC1164	United States	2000	Hetero	database
AY444805	98US_MSC2008	United States	1998	Hetero	database
AB052995	93JP_NH1	Japan	1993	Hetero	database
AB070352	NH25_93JPNH25T	Japan	1993	Hetero	database
AB485652	ID17	Indonesia	1993	n/a	database
GQ477441	569M	Afghanistan	2007	Hetero	database

AF197340	90CF11697	[b]CAR	1990	n/a	database
AF197341	90CF4071	[b]CAR	1990	n/a	database
U51188	90CR402_CAR_E_4002	[b]CAR	1990	Hetero	database

[a]Risk group: Hetero, heterosexual; IDU, injecting drug user; MSM, men who have sex with men; MTCT, mother-to-child transmission; ST, sexual transmission, unspecified type; n/a, not available.

[b]CAR represents Central African Republic.

Supplementary Table S2. Estimated time to the most recent common ancestor of each HIV-1 CRF01_AE cluster.

**CRF01_AE cluster**	**Time to the most recent common ancestor in years (median and 95% credibility interval)**
CRF01_1AE	1995.39 (1994.29, 1996.38)
CRF01_2AE	1990.71 (1989.93, 1991.49)
CRF01_3AE	1993.17 (1991.41, 1994.97)
CRF01_4AE	1993.30 (1992.07, 1994.54)
CRF01_5AE	1994.89 (1993.57, 1996.10)
CRF01_6AE	1993.43 (1991.39, 1995.25)
CRF01_7AE	1998.55 (1997.71, 1999.37)
CRF01_8AE	1987.50 (1986.24, 1988.86)
CRF01_9AE	1990.75 (1989.31, 1992.13)
CRF01_10AE	1998.83 (1998.51, 1999.08)
TH (Thailand HIV-1 CRF01_AE)	1981.45 (1979.92, 1982.89)
PAN (Pandemic HIV-1 CRF01_AE)	1983.73 (1982.70, 1984.73)

Supplementary Table S3. Statistical analysis of the geographic location of CRF01_AE (China is divided into regions).

**Statistic**	**No. of sequences**	**Observed mean (95% CI)**	**Null mean (95% CI)**	***P*-value**
AI		18.3 (17.4, 19.1)	29.9 (28.1, 31.8)	<0.001*
PS		125.6 (124.0, 128.0)	180.2 (174.0, 186.0)	<0.001*
MC (AF)	1	1.0 (1.0, 1.0)	1.0 (1.0, 1.0)	N/A
MC (CF)	3	1.8 (1.0, 2.0)	1.0 (1.0, 1.0)	0.003*
MC (Central of China)	2	1.0 (1.0, 1.0)	1.0 (1.0, 1.0)	N/A
MC (Eastern of China)	50	2.0 (2.0, 2.0)	2.1 (1.5, 3.0)	0.804
MC (Northeastern of China)	43	3.0 (3.0, 3.0)	2.0 (1.1, 3.0)	0.048*
MC (Southwestern of China)	59	3.0 (3.0, 3.0)	2.3 (2.0, 3.0)	0.128
MC (HK)	1	1.0 (1.0, 1.0)	1.0 (1.0, 1.0)	N/A
MC (ID)	1	1.0 (1.0, 1.0)	1.0 (1.0, 1.0)	N/A
MC (JP)	2	2.0 (2.0, 2.0)	1.0 (1.0, 1.0)	0.002*
MC (TH)	134	40.0 (40.0, 40.0)	3.9 (3.0, 5.96)	0.001*
MC (US)	5	2.0 (2.0, 2.0)	1.0 (1.0, 1.0)	0.011*
MC (VN)	33	5.0 (5.0, 5.0)	1.7 (1.0, 2.1)	0.001*

AI, association index.
PS, parsimony score.
MC, monophyletic clade statistic.
95% CI, 95% credibility interval.
*Statistically significant ($P < 0.05$).
N/A, not available because of the observed 95% CI contains the null 95% CI.

Supplementary Table S4. Statistical analysis of the geographic location of CRF01_AE (China is divided into provinces).

Statistic	No. of sequences	Observed mean (95% CI)	Null mean (95% CI)	*P* -value
AI		19.4 (18.5, 20.3)	31.8 (30.0, 33.5)	<0.001*
PS		135.7 (134.0, 138.0)	190.4 (185.9, 194.5)	<0.001*
MC (AF)	1	1.0 (1.0, 1.0)	1.0 (1.0, 1.0)	N/A
MC (CF)	3	1.8 (1.0, 2.0)	1.0 (1.0, 1.0)	0.003*
MC (Beijing)	13	2.0 (2.0, 2.0)	1.2 (1.0, 2.0)	0.077
MC (Fujian)	19	2.0 (2.0, 2.0)	1.3 (1.0, 2.0)	0.138
MC (Hebei)	2	1.0 (1.0, 1.0)	1.0 (1.0, 1.0)	N/A
MC (Hunan)	2	1.0 (1.0, 1.0)	1.0 (1.0, 1.0)	N/A
MC (Guangdong)	10	2.0 (2.0, 2.0)	1.1 (1.0, 2.0)	0.04*
MC (Guangxi)	24	3.0 (3.0, 3.0)	1.5 (1.0, 2.0)	0.009*
MC (Guizhou)	6	1.0 (1.0, 1.0)	1.0 (1.0, 1.0)	N/A
MC (Jiangsu)	5	1.0 (1.0, 1.0)	1.0 (1.0, 1.0)	N/A
MC (Jilin)	8	1.2 (1.0, 2.0)	1.1 (1.0, 1.8)	N/A
MC (Liaoning)	35	3.0 (3.0, 3.0)	1.8 (1.0, 2.4)	0.021*
MC (Sichuan)	2	1.0 (1.0, 1.0)	1.0 (1.0, 1.0)	N/A
MC (Tianjin)	1	1.0 (1.0, 1.0)	1.0 (1.0, 1.0)	N/A
MC (Yunnan)	27	1.5 (1.0, 2.0)	1.6 (1.0, 2.0)	N/A
MC (HK)	1	1.0 (1.0, 1.0)	1.0 (1.0, 1.0)	N/A
MC (ID)	1	1.0 (1.0, 1.0)	1.0 (1.0, 1.0)	N/A
MC (JP)	2	2.0 (2.0, 2.0)	1.0 (1.0, 1.0)	0.004*
MC (TH)	134	40.0 (40.0, 40.0)	3.9 (3.0, 6.0)	0.001*
MC (US)	5	2.0 (2.0, 2.0)	1.0 (1.0, 1.0)	0.006*
MC (VN)	33	5.0 (5.0, 5.0)	1.7 (1.0, 2.2)	0.001*

AI, association index.
PS, parsimony score.
MC, monophyletic clade statistic.
95% CI, 95% credibility interval.
*Statistically significant ($P < 0.05$).
N/A, not available because of the observed 95% CI contains the null 95% CI.

Supplementary Table S5. Statistical analysis of the geographic location of CRF01_AE (China and Vietnam are divided into provinces).

Statistic	No. of sequences	Observed mean (95% CI)	Null mean (95% CI)	*P* -value
AI		20.1 (19.3, 21.1)	32.2 (30.4, 33.7)	<0.001*
PS		143.8 (141.0, 146.0)	192.1 (187.9, 195.9)	<0.001*
MC (AF)	1	1.0 (1.0, 1.0)	1.0 (1.0, 1.0)	N/A
MC (CF)	3	1.8 (1.0, 2.0)	1.0 (1.0, 1.0)	0.003*
MC (Beijing)	13	2.0 (2.0, 2.0)	1.1 (1.0, 2.0)	0.055
MC (Fujian)	19	2.0 (2.0, 2.0)	1.3 (1.0, 2.0)	0.141
MC (Hebei)	2	1.0 (1.0, 1.0)	1.0 (1.0, 1.0)	N/A
MC (Hunan)	2	1.0 (1.0, 1.0)	1.0 (1.0, 1.0)	N/A
MC (Guangdong)	10	2.0 (2.0, 2.0)	1.1 (1.0, 1.9)	0.031*
MC (Guangxi)	24	3.0 (3.0, 3.0)	1.4 (1.0, 2.0)	0.008*
MC (Guizhou)	6	1.0 (1.0, 1.0)	1.0 (1.0, 1.0)	N/A
MC (Jiangsu)	5	1.0 (1.0, 1.0)	1.0 (1.0, 1.0)	N/A
MC (Jilin)	8	1.2 (1.0, 2.0)	1.1 (1.0, 1.5)	N/A
MC (Liaoning)	35	3.0 (3.0, 3.0)	1.8 (1.0, 2.2)	0.018*
MC (Sichuan)	2	1.0 (1.0, 1.0)	1.0 (1.0, 1.0)	N/A
MC (Tianjin)	1	1.0 (1.0, 1.0)	1.0 (1.0, 1.0)	N/A
MC (Yunnan)	27	1.5 (1.0, 2.0)	1.6 (1.0, 2.0)	N/A
MC (HK)	1	1.0 (1.0, 1.0)	1.0 (1.0, 1.0)	N/A
MC (ID)	1	1.0 (1.0, 1.0)	1.0 (1.0, 1.0)	N/A
MC (JP)	2	2.0 (2.0, 2.0)	1.0 (1.0, 1.0)	0.002*
MC (TH)	134	40.0 (40.0, 40.0)	4.0 (2.9, 6.0)	0.001*
MC (US)	5	2.0 (2.0, 2.0)	1.0 (1.0, 1.0)	0.008*
MC (An Giang)	14	5.0 (5.0, 5.0)	1.2 (1.0, 2.0)	0.001*
MC (Bac Giang)	4	2.0 (2.0, 2.0)	1.0 (1.0, 1.0)	0.004*
MC (Hai Duong)	3	1.0 (1.0, 1.0)	1.0 (1.0, 1.0)	N/A
MC (Ho Chi Minh)	10	3.0 (3.0, 3.0)	1.1 (1.0, 2.0)	0.001*
MC (Nam Dinh)	2	1.0 (1.0, 1.0)	1.0 (1.0, 1.0)	N/A

AI, association index.
PS, parsimony score.
MC, monophyletic clade statistic.
95% CI, 95% credibility interval.
*Statistically significant ($P < 0.05$).
N/A, not available because of the observed 95% CI contains the null 95% CI.

Supplementary Table S6. International tourist arrivals to Thailand by Chinese nationals[a].

Year	Number	%Shared[b]	%Δ[c]	Rank[d]
2014	4623806	18.66	-0.29	1
2013	4637335	17.47	66.40	1
2012	2786860	12.47	61.91	1
2011	1721247	8.95	53.38	2
2010	1122219	7.04	44.34	2
2009	777508	5.49	-5.95	4
2008	826660	5.67	-8.87	4
2007	907117	6.27	-4.43	4
2006	949117	6.87	0.22	4
2005	776792	6.72	6.43	4
2004	729848	6.22	0.20	5
2003	606635	6.02	-23.98	5
2002	797976	7.34	-0.42	3
2001	801362	7.91	6.31	3
2000	753781	7.87	-7.35	3
1999	813596	9.40	34.60	3
1998	604472	7.71	33.58	3
1997	452510	6.20		3

[a]Source of data: Immigration Bureau, Police Department, Thailand.
[b]**%**Shared: the proportion of international tourist arrivals to Thailand by Chinese nationals.
[c]%Δ: the difference between the number of current year's and last year's Chinese
[d]Rank: rank China by the source of international tourist arrivals to Thailand.

Supplementary Table S7. Description of the reference near-complete genome sequences for HIV-1 CRF01_AE clusters.

CRF01_AE cluster	Sequence name	Acc. No.	Sampling year	Sampling Country (origin	Reference
CRF01_1AE	05GX034	GQ845124	2005	China	Li, L. *ARHR* **26:** 699-704 (2010)
CRF01_2AE	97VNHCM310	FJ185240	1997	Vietnam	Liao, H. *Virology* **391:** 51-56 (2009)
CRF01_3AE	GZ070123	JX112843	2007	China	Feng, Y. *AIDS* **27:** 1793-1802 (2013)
CRF01_4AE	07CNYN364	KF835542	2007	China	Liu, Y. unpublished
CRF01_5AE	AA033a_wg6a	JX447081	2005	Thailand	Rolland, M. *Nature* **490:** 417-420 (2012)
CRF01_6AE	Fj057	EF036530	2005	China	Huang, H.L. *ARHR* **23:** 569-574 (2007)
CRF01_7AE	YN0235	JX112865	2002	China	Feng, Y. *AIDS* **27:** 1793-1802 (2013)
CRF01_8AE	GD070059	JX112821	2007	China	Feng, Y. *AIDS* **27:** 1793-1802 (2013)
CRF01_9AE	OUR422I	AY358051	1999	Thailand	Tovanabutra, S. *ARHR* **20:** 465-475 (2013)
CRF01_10AE	OUR200I	AY358066	2000	Thailand	Tovanabutra, S. *ARHR* **20:** 465-475 (2013)
